# A history of lesbian politics and the psy professions

**DOI:** 10.1177/0959353520969297

**Published:** 2020-12-08

**Authors:** Helen Spandler, Sarah Carr

**Affiliations:** University of Central Lancashire, UK; University of Birmingham, UK

**Keywords:** lesbian and gay, pathologisation, activism, social movements, feminism, England

## Abstract

This article explores the relationship between lesbian activists and the “psy professions” (especially psychology and psychiatry) in England from the 1960s to the 1980s. We draw on UK-based LGBTQIA+ archive sources and specifically magazines produced by, and for, lesbians. We use this material to identify three key strategies used within the lesbian movement to contest psycho-pathologisation during this 30-year period: from respectable collaborationist forms of activism during the 1960s; to more liberationist oppositional politics during the early 1970s; to radical feminist separatist activism in the 1980s. Whilst these strategies broadly map onto activist strategies deployed within the wider lesbian and gay movement during this time, this article explores how these politics manifested in particular ways, specifically in relation to the psy disciplines in the UK. We describe these strategies, illustrating them with examples of activism from the archives. We then use this history to problematise a linear, overly reductionist or binary history of liberation from psycho-pathologisation. Finally, we explore some complexities in the relationship between sexuality, activism and the psy professions.

## Background

Challenging the psycho-pathologisation of homosexuality was a key focus of struggle for the gay liberation movement, and a touchstone issue for the anti-psychiatry movement during the 1960s and 1970s, especially in the US and UK. The success of the campaign to remove homosexuality as a mental illness from the *Diagnostic and Statistical Manual* (DSM) for psychiatric diagnoses is well-documented (e.g. Bayer, 1987; Drescher, 2015; Drescher & Merlino, 2007; Minton, 2002). In addition, feminist critiques of the medicalisation and pathologisation of women’s bodies, desires and sexualities is well-rehearsed (e.g. Cryle & Downing, 2009; Kitzinger & Perkins, 1993; Nicolson, 1993; Ussher, 1989). However, we know less about the different activist strategies used to contest pathologisation in the UK context, specifically in relation to lesbian sexuality (King & Bartlett, 1999). Recent research has started to address this gap, especially in relation to reclaiming some feminist psychologists as early activists and pioneers of LGBTQIA+^1^ or Queer affirmative practice (e.g. Hubbard, 2019; Hubbard & Griffiths, 2019).

Whilst the emancipation of homosexuality from the “registers of mental pathology” helped secure the conditions of possibility for lesbian and gay rights, the historical privileging of the 1970s US de-classification campaign has “eclipsed more nuanced challenges to psy authority and obscured more complex activist engagements with sexuality, mental illness, and the psy professions” (Lewis, 2016, p. 84). Therefore, an analysis of activist strategies in relation to the psy professions is warranted, to explore activism beyond declassification (and decriminalisation) and campaigns. Moreover, a focus on the UK context is important since there is less written on this subject, especially regarding women’s activism. Historiography in this field has typically focused on white, cisgender, gay male actors in US context (Hegarty & Rutherford, 2019). Notable exceptions include: Rebecca Jennings’ (2008) detailed account of lesbians and psychiatry in the *Journal of British Studies*; Alison Oram’s (2007) chapter in *The Permissive Society and Its Enemies*; Oram and Turnbull’s (2001) commentaries in their *Lesbian History Sourcebook*; and Katherine Hubbard’s (2019) history of how psychologists used the Rorschach ink blot test to contest the pathologisation of lesbians.

This research forms part of a broader “hidden from history” project committed to constructing more comprehensive histories of marginalised and oppressed communities (Duberman et al., 1989). More specifically, it contributes to recent Queer/LGBTQIA+ and feminist histories of British psychology, psychiatry and the psy professions which complicates overly simplistic or binary histories of “progress, stagnancy, or regression” (Hubbard & Griffiths, 2019, p. 941; see also Hegarty & Rutherford, 2019; Hubbard, 2019). This article complements this body of work by focusing primarily on activists outside the psy disciplines, rather than psy professionals who tried to challenge pathologisation “from within”. However, we recognise that such a neat outside/inside boundary is not always easy to sustain in practice (Hubbard, 2019).

## Identifying political strategies: Methodologies and limitations

We draw on findings from our “bottom-up” study of UK-based LGBTQIA+ archives investigating what happened to lesbian and bisexual women in the British mental health system from the 1950s until 1990 (when the World Health Organisation officially declassified homosexuality as a mental illness). Our broader research found small numbers of women who were subjected to a variety of experimental psychological, psychiatric and psychotherapeutic “treatments” for their sexuality, including aversion therapy (Carr & Spandler, 2019; Spandler & Carr, 2020).

For this article, we identified and analysed magazines and other ephemera produced by, and for, lesbian communities from the 1960s to 1990, alongside analyses and commentaries on the work of individuals and groups who produced these publications. There were few complete collections in any of the archives, with various editions being scattered across different libraries and collections.^2^ However, we were able to locate and explore every issue of the following: *Arena Three,* a magazine for lesbians (1964–72); *Come Together*, a magazine of the Gay Liberation Front (1970–73); and the *Lesbian Information Service Newsletter* (1987–9). We also located most issues of *Sappho* (1972–81) and the *Revolutionary and Radical Feminist Newsletter* (1978–89), and several issues of *Lesbian Express* (1977–9). Whilst not exhaustive, these resources provided us with sufficient material to understand the shifting politics, positions and strategies of lesbian activism during this period.

The following sections identify three key strategies deployed by lesbian activists to challenge the psycho-pathologisation of homosexuality, highlighting continuities and ruptures in the strategies deployed. These often relate to different approaches taken to gay and women’s liberation, as well as attitudes towards the “psy” disciplines (psychology, psychiatry, psychotherapy and related professions). For the sake of clarity, and to help crystallise our analysis, we have categorised these chronologically into three main strategies: collaborative, oppositional, and separatist. This categorisation is used as a heuristic device to identify the prominent approaches lesbian organisations took towards the psy professions during these three decades. The orientations we identify roughly map onto broader lesbian activist strategies in the UK during this time (e.g. Hamer, 1996) but the primary purpose of this article is to explore how these strategies “played out” in relation to the psy disciplines, a history which is lesser known. We identify key organisations and publications that articulate the strategy identified and give some examples of their activism.

By taking a “broad brush” approach to this history, we inevitably risk oversimplification and overgeneralisation. In reality, of course, the evolution of activism was a complex process with significant overlaps, both in the strategies employed and the activists who deployed them, rather than a linear sequence of clearly defined political strategies. For example, some activists adopted several diverse positions and tactics and were involved in more than one of the strategies discussed. In addition, LGBTQIA+ history is itself deeply “entangled” (Hubbard & Griffiths, 2019), as are the psy professions. Whilst our focus on lesbian activism is important in filling historical gaps and erasures, inevitably it risks other exclusions. For example, it is primarily a white, middle class, cisgendered and Anglocentric history.

Moreover, our reference to lesbian politics is not intended to exclude bisexual or other sexualities who may have been involved in activism. There was a more limited range of sexual and gender identities available during this time and lesbian was usually the preferred terminology and identity used by activists. It helped to highlight women’s specific experiences which were too often subsumed under the overly medicalised term “homosexuality” which also, like “transvestitism”, tended to refer to men. Moreover, despite some scholars suggestions that we have entered an era of “post lesbian” discourse, the term held, and to some degree continues to hold, deep meaning, and material consequences for some women’s everyday lives, and their political mobilisation (Forstie, 2019; McNaron, 2007). These reasons are also why we refer to the “lesbian and gay” movement, rather than the more contemporary and inclusive “LGBTQIA+” movement, even though activists may not have all been exclusively lesbian, gay or cisgendered. We hope further research will be undertaken to develop a more inclusive, intersectional and international history.

## Collaborative strategies: 1960s

This strategy was most clearly articulated by the early lesbian magazine *Arena Three* (1964–72), produced by the Minorities Research Group (MRG), a lesbian pressure group. Whilst there are examples of lesbian activism prior to this period, *Arena Three* (A3) is generally regarded as the first lesbian magazine in the UK and the MRG as the “first explicitly lesbian social and political organisation in Britain” (Oram, 2007, p. 63). It was established at a time when lesbian visibility in the UK was “close to non-existent” (Jennings, 2013, p. 136) and media representation was “infrequent” or “overwhelming negative” (Jennings, 2013, p. 150): “if lesbians were to be mentioned at all, it was only by way of the smoking room snigger or the psychiatric ‘case history’” (*Arena Three*, 1969, 6.12, p. 1). The collaborative strategy of activists involved with MRG/*Arena Three* broadly mirrors the lesbian magazine *The Ladder* (1956–72) and the *Daughters of Bilitis* organisation in the US (e.g. Esterberg, 1994; Soares, 1998; Vigiletti, 2015) with some distinctive features and differences (see Hubbard, 2020).

*Arena Three*/MRG activists did not see the psy professions as primarily responsible for lesbians’ predicament, but rather as potential allies to challenge it. For example, the famous quote from Freud that homosexuality should not be seen as illness was often used in their promotional literature. Whilst their subscribers and supporters may have come from a relatively broad social base, it was initiated by a small group of formally educated, middle-class lesbians. Perhaps because of their background, they had greater access to, and were possibly positively influenced by, medical and psy discourse (Hubbard, 2020). As a result, they initiated dialogue with the psy professions, and discussion about medical, psychological and psychiatric understandings of lesbianism often featured in early issues of the magazine. For example, the first few issues included debate about the “causes” of lesbianism involving binary debates about whether it was “acquired” or “inborn”. This reflected the broader debate on aetiological theories and clinical definitions (British Medical Association, 1955) but also the need to shift the prevailing discourse away from homosexuality as a moral or criminal problem. Although the sociologist Mary McIntosh wrote a short piece arguing against a binary view of the causes of homosexuality in 1964 (*Arena Three*, 1964, 1.5, pp. 4–6), there were limited alternative or non-binary discourses available at that time (Hubbard, 2020). For example, lesbian sexuality was rarely seen as a positive choice (Hamer, 1996). There was also a willingness to seek assistance from the psy professions to help women understand, explain and resolve their situation, not necessarily to change their sexual orientation but to assist them to ascertain if they were “really” lesbian and, if so, to help them accept their sexuality. Getting psy professionals “on side” was viewed as an important political strategy, the idea being that if lesbians could be shown to be psychologically “normal”, they could be better understood and accepted by wider society.

Whilst *Arena Three* and MRG could certainly be described as feminist, they did not see the pathologisation of lesbians as necessarily interlinked with other forms of oppression. Their strategy was “assimilationist” as it didn’t aim to challenge the wider structures of society, or the psy disciplines, but to get a more elastic and inclusive view of normative sexuality, that included female homosexuality. As the overall strategy was to make lesbians “acceptable”, activists engaged in socially respectable forms of activism. Letters from key MRG activists, such as Esme Langley, suggested they engaged in various attempts to politely engage with more established authorities and ally organisations. For example, in one letter Langley invites a prominent individual for a discussion over tea and sandwiches. Activists emphasised the importance of lesbians being “discrete” and “respectable decent women who just happened to be lesbians” (Hubbard, 2020, p. 89). As a result, they often distanced themselves from lesbians who drew attention to their sexuality, or who did anything that might be considered “perverted” or challenged prevailing gender norms. Moreover, many MRG members were married, and the organisation even had an official policy that husbands’ signatures were required to confirm their wives could join.

Engagement in research studies was an important part of activists’ strategy to challenge pathologisation. The idea that homosexuality was a mental disorder rested on the idea that lesbians and gays were not only different to heterosexuals, but also mentally unstable and neurotic. Many psychiatric and psychological studies about homosexuality up to this point had used research subjects who were in the psychiatric system and therefore already deemed to be mentally unwell, resulting in a skewed view of the “homosexual psychology”. To rectify this, activists were keen to make sure research about homosexuality included “normal” or “non-patient” populations who were not in the mental health system. As a result, *Arena Three* actively encouraged lesbians to volunteer in psy studies as “normal” research subjects.

Activists had regular contact with several psy professionals who they sometimes approached for advice and assistance and, in turn, psy professionals approached the MRG for help recruiting “subjects” for their research studies, including June Hopkins, Charlotte Wolff, Eva Bene, and F.E. Kenyon. Many of these psychologists turned out to be lesbian or bisexual themselves, even though they weren’t necessarily “out” at the time, especially within the profession. This “blurs the boundary” between lesbian activists and the psy professionals being separate categories (Hubbard, 2020, p. 21). At the same time, activists acted as gatekeepers for researchers to access lesbians from the wider community. There are several “acknowledgements” to the MRG in reports of studies about homosexuality in mainstream medical and psychiatric journals, such as the *British Journal of Psychiatry*. One activist, Cynthia Reid, felt she had to reassure readers who might be worried that participating in psy research implied their sexuality was a sickness: “reputable psychiatrists and psychologists would not attempt to treat or cure their sexuality, but may be required to treat people who are depressed or depressed as a result of their sexuality in a predominantly heterosexual society” (*Arena Three*, 1965, 2.4, pp. 10–11).

Rather than being beholden to psychiatric or psychological knowledge, the MRG “sought to bring psychiatry in line with their own analysis” (Oram, 2007, p. 69). For example, Hubbard has detailed how June Hopkins, a psychologist allied to MRG and *Arena Three*, used the Rorschach inkblot test to show that lesbians were no more neurotic than heterosexual women and might even have positive psychological characteristics (such as independence). Whether activists believed that the research studies could reveal anything true or meaningful about their sexuality is unclear. However, even if activists didn’t fully agree with the way the research was carried out, they felt it was better to actively engage in studies that would likely happen anyway, so they could attempt to influence their results. They hoped it would help increase knowledge and understanding of their predicament, which in turn could be used to “improve the public image of lesbians, explode myths and reconcile the general public and psychiatrists with the fact of homosexuality as a way of life” (*Arena Three*, 1965, 2.8, p. 9).

In hindsight, whilst this strategy may have helped “turn the tide away from pathologisation” (Hubbard, 2020, p. 84), there were certainly risks with using psy-centred research methods. It tended to assume binary distinctions and categorisations (such as heterosexual/homosexual) and some of the results of the studies were “decidedly mixed” and didn’t necessarily concur with activists’ hopes or expectations (Hubbard, 2020, p. 73). In addition, some activists were wary of any research which might suggest that lesbians were different, as it might be used to signal pathology (this was seen as especially problematic in the US context). The collaborative strategy also resulted in women being involved in some quite bizarre and invasive clinical research studies. For example, Diana Chapman (1985) of the MRG, who volunteered to be a participant in one study at the Maudsley psychiatric hospital in South London, described being subjected to psychological, hormonal and physical tests to discover if there were any measurable anatomical and physiological differences between lesbians and straight women.

Whilst these kinds of studies may be considered unethical or degrading by today’s standards, they were not only used to challenge pathologisation, they were also subverted, at least to some degree. For example, Chapman suggested that opportunities to engage in these studies offered a rare chance for lesbians to get together and were experienced like an “uninhibited party” (1985; see also Jennings, 2008). Moreover, activists were not completely naïve or uncritical about engaging with psy-centred research studies. They adopted “a stance of critical distance” and were generally “ambivalent” towards medico scientific research, seeing it very much as a “means to an end” (Jennings, 2008, pp. 901–902). In addition, whilst they collaborated with selective psy professionals who were supportive of their situation, they were highly critical of any professionals or research studies that took a negative view of lesbians. For example, *Arena Three* included many scathing, and often satirical, reviews of books or articles that misunderstood or pathologised their sexuality.

By the latter half of the decade activists expressed increasing “disillusionment” and “disaffection” towards the psy establishment (Jennings, 2013, p. 147). This shift was probably due to the growing influence of the emerging gay and women’s liberation movements which enabled a less apologetic and more confident lesbian identity and community which was more willing to define itself regardless of, or even in opposition to, psy-science. As a result, some activists from *Arena Three*/MRG started to make more alliances with the emerging gay liberation and women’s liberation movements and engage in more oppositional strategies to challenge their oppression.

## Oppositional strategies: 1970s

By the early 1970s, the rise of counter-cultural social movements of the 1960s and 1970s helped foster a more radical and oppositional lesbian and gay politics in the UK. Activists turned away from psychological and psychiatric theories to try and understand themselves and challenged the idea that psy professionals had any specialised knowledge they could use to help them “put their case to a wider society” (Jennings, 2008, p. 904). Instead they turned to the collective action of lesbian and gay people themselves (Ettorre, 1985; Power, 1995). In addition, activists turned away from trying to understand the homosexual or lesbian “condition”, towards challenging their *social oppression*. They expressed less concern about the “causes” of their sexuality and were more likely to embrace it as a positive choice.

In contrast to previous activism that tried to gain societal acceptance by proving lesbians were just as “normal” as heterosexuals, this strategy was characterised by a more radical, assertive and visible celebration of sexual difference. In other words, the focus moved to a revolutionary transformation of society rather than an adjustment to it; challenging normality rather than normalisation; and emphasising protest rather than collaboration (Power, 1995; Weeks, 1977). As such, activists drew on Marxist, anarchist and anti-imperialist ideas and expressed greater awareness of the links between sexuality, gender, social class and race (Hamer, 1996; Hubbard, 2019). Activism involved active public opposition to “respectable” mainstream society and more importantly, for our purposes here, to the psy professionals. Activists started to see the psy professions, if not individual psy professionals, as more of an obstacle to gay liberation, by enforcing “straight” societal norms. The GLF’s position on psychiatry and the psy professionals is similar to that expressed by some US gay liberation activists such as Frank Kameny (1972), who, like Mary MacIntosh in *Arena Three*, was voicing critique of collaborationist strategies in *The Ladder* magazine in the mid-1960s. In the UK this orientation was represented by *Come Together: A journal for the gay community* (GLF) (1970–3) and *Sappho: A women’s liberation magazine* (1971–81). We’ll look at these in turn.

### GLF/*Come Together*

The Gay Liberation Front (GLF) represented a “shift away from an earlier rapprochement” (Jennings, 2008, p. 904) to a more confrontational stance towards the psy professions, especially psychiatry. The 1971 GLF Manifesto explicitly named psychiatry as a source of oppression and the “Counter Psychiatry Group” (CPG) was one of their first action groups. The CPG published a pamphlet, *Psychiatry and the Homosexual: A brief analysis of oppression*, which demanded that “all gay people in mental institutions by reason of their homosexuality should be freed and given reparation” (*Come Together*, 1970, Issue 4, p. 6). They clearly stated that homosexuality is not a mental disorder, or arrested development, and does not require treatment or therapy. Instead activists suggested psy professionals refer their homosexual clients to the gay liberation movement. Whilst membership of the CPG, like the GLF, wasn’t large in number, it engaged in the most prolonged activism throughout life of the organisation and, according to Power, was “one of the most influential groups in the development of gay politics and gay pride” (Power, 1995, p. 90).

Whilst the GLF was often male dominated, and this was a cause of later friction in the movement, several key activists in the CPG were lesbians, for example, Jackie Forster, who had been involved in *Arena Three* and the Campaign for Homosexual Equality; and two of the founders were Elizabeth Wilson and her then partner, the sociologist Mary McIntosh, both of whom had also been MRG members.^3^ Wilson and McIntosh called the first meeting of the CPG in their house. Wilson played a leading role in the group, drawing on her experience as a “very unhappy psychiatric social worker who saw how homosexuality and women were treated in the [psychiatric] system” (Power, 1995, p. 91). She recalled hearing “horror stories about people being treated with shocks and aversion therapy” (Wilson, in Power, 1995, p. 92). It is unclear how many members of the CPG had actually experienced the psy system, as patients, but Wilson did recall “someone [presumably male] coming along who had had aversion therapy” (Wilson, in Power, 1995, p. 92). Unlike male homosexuality, lesbian sexuality wasn’t directly criminalised, so women were less likely to be offered these treatments as a “softer” alternative to prison (Dickinson, 2013; King & Bartlett, 1999). Earlier lesbian activists were aware of this treatment, and it was referred to disparagingly in early issues of *Arena Three,* although it wasn’t a key focus of their activism. But the CPG actively opposed *any* form of psy treatment, especially aversion therapy, and Wilson used her insider knowledge of the psy profession to identify specific targets for GLF’s activism. Activists were clearly influenced by the broader anti-psychiatry movement as they were familiar with the work of people like R.D. Laing, whose ideas were popular in social work training and circulated within wider counter-cultural movements at this time (Jennings, 2008; Power, 1995). This, alongside the knowledge that gay men were being subjected to aversion therapy, and other stories of psy oppression that probably circulated in gay and lesbian bars and groups, influenced their antagonism towards the psy professions (Jennings, 2008).

**Figure 1.**
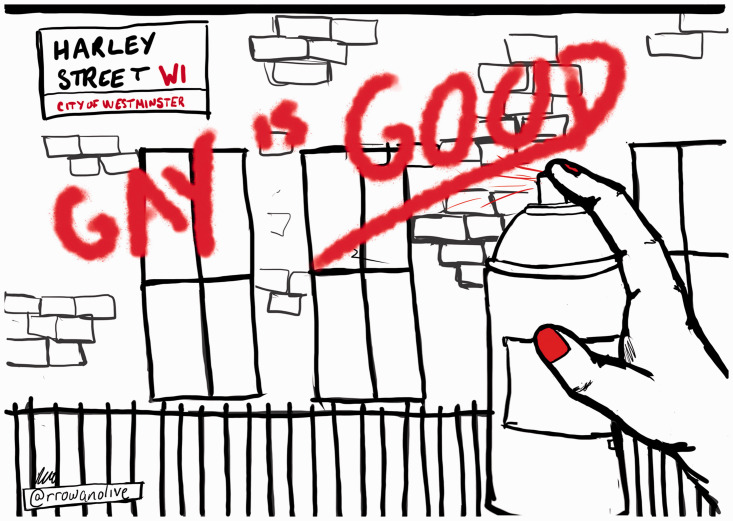
‘Gay is Good’ by Rachel Rowan Olive

Inspired by the civil rights movement, the GLF adopted a strategy of non-violent direction action and held awareness raising “teach-ins” about psychiatric treatment and other forms of discrimination and oppression (Forster, 1985). In February 1971 activists protested in bookshops about the sale of David Reuben’s *Everything You Always Wanted to Know about Sex – But Were Afraid to Ask,* due to its pathologising chapter on homosexuality and female homosexuality being included with prostitution. In the summer of 1971 members of the CPG initiated a series of set piece “hit and run” demonstrations known as “zaps”. A women’s issue of *Come Together* advocated “the mounting of attacks on institutions that specifically oppress gay people. If we are serious, we should make it impossible for places like the Maudsley and the Portman Clinic [which were known to hospitalise lesbian and gay people and ‘treat’ their ‘sexual deviation’] to exist” (*Come Together*, 1971, Issue 7, p. 5). In June 1971 activists organised a demonstration in Harley Street against what they framed as bourgeois psychiatry and its oppression of gay people. The evening before the demo, a small group of activists spray-painted the whole length of Harley Street with slogans like “gay is good” (see Figure 1) (Power, 1995, p. 96).

Jackie Forster recalled attending a GLF meeting and finding out about aversion therapy and the next day getting involved in a “zapping” of the bank which held the account of the Maudsley psychiatric hospital which was known to hospitalise lesbian and gay people and “treat” their sexuality (Forster, 1985). Members of autonomous lesbian groups also worked alongside the GLF. For example, women from the Leeds Lesbian Group and the Bradford Gay Women’s Group joined forces with CHE and the GLF to gate-crash a medical conference on “psychosexual disorders” at Bradford University which discussed homosexuality, transsexuality and transvestitism (*Sappho*, 1974, Issue 3.6). There was also a short-lived autonomous group of GLF women, the Red Lesbian Brigade. Members of this group spray-painted and leafleted hospitals like the Tavistock and Maudsley in London to specifically protest against aversion therapy. The leaflets were in the form of a letter to psychiatrists and psychologists that said:

Further to our slogans painted outside, the enclosed explanatory information may be of interest to you.

WE ARE NOT SICK. WE ARE NOT ABNORMAL. WE ARE NOT IMMATURE.

Stop making a fat living out of saying we are.

WE ARE STRONG. WE ARE BEAUTIFUL. Power to the patients, we don’t need you!

In anger

Red Lesbian Brigade. (in Power, 1995, p. 124)

### Women’s liberation and *Sappho*

It appears that lesbianism was “first raised in a big way” as a concern for the women’s liberation movement in the UK at the Skegness Women’s Liberation conference in 1971 (Walter, 1980, p. 31). However, it was still seen as a marginal issue within the wider women’s movement (Hamer, 1996). According to Walter, once lesbians had gained some acceptance in the women’s movement, “the parting of the ways [between gays and lesbians in the GLF, formalised in 1972] was perhaps inevitable” (Walter, 1980, p. 32). *Sappho* magazine (1972–81) was the culmination of lesbians’ increasing desire to organise autonomously, but alongside both the women’s and gay liberation movement. It represented the convergence of gay and women’s liberation politics during the early 1970s and, more importantly, filled a gap between the two. Arguably it picked up where *Arena Three* had left off, involving some of same people, such as Diana Chapman, but was more explicitly feminist and closely aligned with the Women’s Liberation Movement.

The women’s movement was becoming increasingly critical of “patriarchal” medicine, and these critiques influenced and shaped lesbian activist responses to psychiatric authority (Lewis, 2016). Unlike *Arena Three*, there are very few examples in *Sappho* of readers’ letters and articles expressing a wish to be “normal”. Instead there was a more positive assertion of lesbian visibility. For example, whilst *Arena Three* often published people’s letters and articles anonymously, *Sappho* tended to print people’s full names, unless authors specifically requested otherwise. The name itself, *Sappho*, was much more “out” than *Arena Three.*^4^ This was a sign of an increasing politicisation of sexuality and a growing positive consciousness, pride and confidence. *Sappho* was edited by Jackie Forster, who had been a member of both CHE and the GLF and may have influenced its increasingly critical attitude towards the psy professions. For example, a BBC Radio 4 Woman’s Hour programme about lesbians that ended with a sympathetic view from a female psychologist who had been an ally of *Arena Three*/*MRG* activists was heavily criticised in a review published in *Sappho*. The reviewer asked: “why does the media insist on a psychiatric follow-up anyway?” and suggested “a policy of non-co-operation might be advisable” (*Sappho,* 1974, Issue 3.2, pp. 18–19). Whilst individual psychologists who were supportive of the lesbian cause were still regarded with “much affection and esteem”, they were considered a paradox in a profession that was increasingly derided (*Sappho*, 1977, Issue 6.1).

Their editorial stance resulted in psy professionals writing letters to the magazine criticising negative attitudes expressed towards them. For example, one (gay) psychologist wrote that they “do not try to cure or indoctrinate” but can actually “speed up a person’s adjustment and acceptance of a gay life” (*Sappho*, 1974, Issue 2.12, p. 9). Later, another letter from a lesbian trainee psychiatrist suggested that people often end up seeking help from psychiatry because of the absence of a supportive gay community, and argued that it was “up to women to support each other more … unite and fight [and not] merely grumble about psychiatrists” (*Sappho*, 1978, Issue 6.6, p. 9). Despite these examples, the overall tone of the magazine was increasingly critical of the psy professions throughout the 1970s. For example, Forster issued a scathing response to a letter which defended psychiatrists as “generally sympathetic” to homosexuals. Her response illustrates the shift to an oppositional stance to the psy professions:

[T]his surely, is the very reason for our distrust of them. We have … no need of sympathy, and when it is offered unnecessarily it is dangerous, inasmuch as it tends to create precisely the condition its purports to alleviate. … If psychiatrists are genuinely concerned for our welfare they would do better to concentrate their remedial skills on the really maladjusted and anti-social, the queer bashers and bigots who harass us. (*Sappho*, 1978, Issue 6.8, p. 3)

*Sappho*, Forster declared in an editorial, was “not a friend to psychiatry and all its works” (*Sappho*, 1977, Issue 6.1). Similarly, Sheila Cameron’s review of *Society and the Healthy Homosexual* by George Weinberg claimed that psychiatrists had “caused untold harm by teaching individuals to detest themselves [and]. … were reactionary upholders of public morality” (*Sappho*, 1975, Issue 4.1, pp. 22–3). In addition, in stark contrast to *Arena Three,* which saw psy professionals as potential allies in the struggle again public ignorance, a *Sappho* editorial claimed that they contribute to wider heterosexual prejudice and might be actively harmful to lesbians: “the modern gay woman … is so burdened with psychiatrists, social workers and counsellors that she is encouraged to put a premium on self-pity, which is far more destructive of personality than heterosexual antagonism” (*Sappho*, 1977, Issue 5.9, p. 3).

It is interesting to note that whilst *Arena Three* included several positive accounts of women’s experiences of seeing doctors, psychiatrists and psychologists, *Sappho* didn’t include many actual examples of negative treatment experiences, which were largely assumed. Stories of negative psy treatments were not expressed, or at least not published, until the next stage in the movement.

## Separation strategies: 1980s

The separatist strategy of the 1980s was characterised by a radical lesbian-feminist political orientation and influenced by broader separatist politics (Hamer, 1996). In part, this was a product of disagreements and splits within the broader Gay Liberation movement where some women felt their concerns and voices were marginalised. Some commentators have suggested that radical lesbian feminist activists were more likely to be younger working-class women who “were tired of being lectured by the middle class women” (Power, 1995, p. 133). This strategy is represented by several “women-only” magazines such as the *Lesbian Information Service Newsletter* (LISN), later re-named the *International Lesbian Information Service Newsletter*; *Lesbian Express* (Manchester); and the *Revolutionary and Radical Feminist Newsletter* (Leeds).

**Figure 2.**
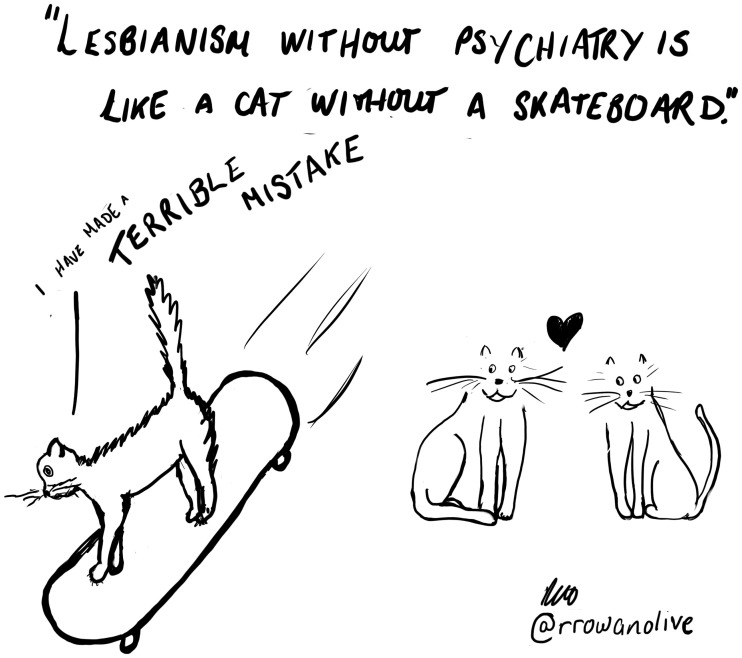
‘Skateboard’ by Rachel Rowan Olive.

These groups went further than *Sappho* in demanding separate, not just autonomous, organisation from the broader gay movement, as well as the psy professions. Radical lesbian feminists tended to be even more critical of the psy professionals than the oppositionists. This was similar to the US context, where radical lesbian feminists offered “perhaps the most consistent and vocal excoriations of the therapeutic professions” (Lewis, 2016, p. 103). In the UK, an article by Isobel Irvine (*Lesbian Express*, June 1979, pp. 3–6) argued that lesbians should “steer clear of psychiatrists at all costs” as they are a “modern form of witchcraft” and the “deadly enemy”. Psychiatrists, she said, “have the power to put you away for life … Once in their hands you’ve had it and will be scarred for life.” Similarly, in a later article, Dinah Mite refers to the psychiatric practice of psychosurgery as primarily done to women as a form of “patriarchal social control and oppression” (*Revolutionary and Radical Feminist Newsletter*, Summer 1981, pp. 6–11). This separatist political orientation is neatly summed up by lesbian feminist activist and scholar Betty Ettore: “In popular feminist folklore, there is an expression, ‘A woman without a man is like a fish without a bicycle’ … one might also say: ‘Lesbianism without psychiatry is like a cat without a skateboard’” (see Figure 2; Ettorre, 1985, p. 421).

Ettorre referred to psychiatry as “Psych/Atrophy: a form of a male-instigated degradation” (Ettorre, 1985, p. 425) and psychiatric practice as specifically “anti-feminist and anti-lesbian”, aimed at “eradicating lesbianism by enforcing the compulsory heterosexist order” (Ettorre, 1985, p. 426). She argued that the psychiatric profession is harmful as it “obstructs, opposes and contradicts the feminist process of Self-Healing” (Ettorre, 1985, p. 424).

This approach to activism was a blend of the women-centred supportive focus of the early MRG/*Arena Three* activists and the anti-psychiatry of the early gay liberationists. Whilst the radical lesbian feminists seemed to be just as antagonistic towards the psy professions as the oppositional activists, they were less focused on directly opposing the psy professions (although they did engage in direct action in relation to other issues they saw as reinforcing patriarchal oppression, such as pornography and sado-masochistic sexual practices). These activists articulated a “very different affective politics”, in contrast to the “adjustment” advocated by the assimilationists or the “pride” advanced by gay liberationists (Lewis, 2016, p. 107). They seemed more focused on providing alternative forms of information and spaces for women to support each other, separate from men and the psy professions.

For example, the *Lesbian Information Service Newsletter* (LISN) included a regular section entitled “Lesbian Tales” for lesbians to write about their experiences as lesbians. It included several examples of women being mistreated and abused in psychiatric hospitals (e.g. *Lesbian Information Service Newsletter [LISN]*, 1987, No. 5, pp. 16–18; LISN, 1987, No. 4. pp. 11–12). Women recalled their experiences of being subjected to a wide variety of different degrading and unhelpful treatments, including group therapy, deep insulin coma treatment, electroconvulsive therapy (ECT), hormone treatment to increase their femininity, and hypnosis. They also reported being subjected to insults and intrusive questions about their sexuality by psychiatric hospital staff, and some reported having to fake heterosexuality in order to be discharged.

By the late 1980s, lesbian feminist activism turned back towards the psy professions. Not just “in collaboration with” (like *Arena Three*/MRG) or just “in opposition to” (like GLF/*Sappho*), this activism was, paradoxically, “within *and* against” the psy professions. Preceding activist efforts had helped create a space for “out” lesbian psychologists who developed a lesbian feminist movement within British psychology which was critical of psychology. Central to this movement was the psychologist Celia Kitzinger, who had been a psychiatric in-patient and received psychological “treatment” for her sexuality as a teenager (Kitzinger, 1987). A separationist strand of lesbian activism continued into the 1990s. For example, a letter in *Lesbian London* by the psychologist Rachel Perkins and Jackie Bishop about lesbians struggling with mental health problems proclaimed: “we are not interested in developing new therapies, but in developing feminist theory and practice so our communities can accept us all” (*Lesbian London*, Feb. 1993, p. 5). Insider activism by lesbian (and gay) psychologists eventually led to the establishment of a Lesbian and Gay section within the British Psychological Society, and, even later, within the Royal College of Psychiatry (Hubbard & Griffiths, 2019). This history, at least within British psychology, has been documented elsewhere (Burman, 2011; Wilkinson & Burns, 1990).

## Complicating histories of activism and the psy-professions

This section uses our research to complicate a linear or binary history of the relationship between activism, the psy professionals and sexual liberation. For example, we try to avoid the tendency to endorse either a “triumphant” story of homosexuality’s emancipation from psycho-pathologisation, or an alternative “tragedy” story of the incorporation of LGBTQIA+ liberation through continued pathologisation (Hegarty & Rutherford, 2019). Instead of merely reproducing one of these narratives, we try to chart a more complex history of activism and the psy disciplines. We do this by introducing some contradictions and complexities into our understanding of the various strategies deployed by activists.

The early collaborationist stance was anathema to later radicals – both gay liberationist and radical lesbian feminists – as well as contemporary Queer and other radical LGBTQIA+ activists. By modern activist standards, it may seem conservative – quaint at best, reactionary at worst – in endorsing heteronormative, Western, white, middle-class notions of respectability and normality. Activists did seem to adopt a distinctly white middle-class feminist perspective, with a stark lack of “intersectional” awareness (Hubbard, 2020). At the same time, this early lesbian activism probably helped at least some lesbians gain a more positive view of themselves, get support from others, gain self-respect and develop a sense of a community. In other words, it enabled the emergence of an “identity, a culture and a movement” (Soares, 1998, p. 47). It helped transform lesbians from a hidden marginalised group into a “proud and vocal part of both the gay and women’s liberation movements” (Soares, 1998, p. 47) and “arguably reconﬁgured the relationship between lesbians and medico-scientiﬁc discourse” (Jennings, 2008, p. 901). For example, rather than completely rejecting psy science, some activists utilised some psy-centred discourse and practice to challenge pathologisation as a form of “activist” or “emancipatory” science (Hubbard, 2017; Pettit, 2011). Indeed, some recent scholarship has reclaimed this work as early examples of LGBTQIA+ “affirmative psychology” (Hubbard, 2020; Hubbard & Griffiths, 2019). Moreover, Alison Oram suggests that, despite its conservative reputation, *Arena Three* and the MRG had all the characteristics of a progressive new social movement and “created an innovative lesbian politics well before the appearance of Gay Liberation” (Oram, 2007, p. 63). It also paved the way for future activists and more radical action.

Yet assimilationist strategies which appealed to supposedly objective “science” and to psy professionals to affirm homosexuality as “normal” yielded mixed results (Lewis, 2016). For example, it has been argued that establishing homosexuality as “normal” left other forms of identities, experiences and behaviours still at the mercy of psy disciplinary regimes of classification and treatment – for example, “Gender Identity Disorder” (Kunzel, 2017; see Bryant, 2006, for a detailed history of this diagnosis). In addition, this strategy arguably strengthened the damaging binary distinction between “normal” and “abnormal” lesbians and implied that lesbians with mental health problems should be strategically marginalised for the greater cause, what has been called a type of “strategic sanism” (Carr, 2019). In the longer term, this may have done a disservice to many LGBTQIA+ people who also have mental health problems, either related or unrelated to their sexual and gender oppression.

In addition, there is some suggestion that the more oppositional strategies of gay liberation activists may have been counter-productive in hardening the views of some psy professionals. For example, according to a survey of behaviour therapists, respondents who had attended a gay liberation meeting said they were less willing to treat homosexuals for something *other than their sexuality* and were less likely to believe homosexuals could be happy and well adjusted (Davison & Wilson, 1973). Whilst survey results are always difficult to interpret, especially establishing any cause and effect relationship, the authors concluded that “perhaps gay liberationists should examine their own tactics when dealing with the professional community” (Davison & Wilson, 1973, p. 694). Notwithstanding this caveat, the oppositional activists certainly played an important part in declassifying homosexuality as a mental illness, raising awareness about harmful and ineffective practices like aversion therapy, and spearheading many new LGBTQIA+ groups, activists and communities (Power, 1995).

Separatist strategies can be criticised as being essentialist or engaging in unnecessary fragmenting of what should have been a broader progressive movement for sexual and gender liberation. In particular, lesbians of colour were reportedly sceptical of the revolutionary potential of lesbian separatism (Forstie, 2019). Yet, as Power argues, “it should be remembered that those politics arose out of a more than justified anger” at how women were treated within these organisations (1995, p. 242). In addition, it is tempting to see this approach as reactionary, especially their wider opposition to S&M and pornography, and what they considered safe for some women might have been experienced as unsafe, or at least unwelcoming, for other women (such as trans and bisexual women). Despite this, these spaces may have allowed some women’s experiences of psychiatric oppression to be articulated, perhaps for the first time. More generally, we need to bear in mind that hindsight bias can underestimate the risks that activists took in bringing about important, if limited and less-than-ideal, social changes (Hegarty & Rutherford, 2019).

More generally, whilst the medicalisation, psychologisation and psychiatrisation of homosexuality is well-cited in LGBTQIA+, Queer and critical mental health scholarship, it is not clear how influential, monolithic or damaging these discourses and practices were. Indeed, our wider research suggests that many psy practitioners did not endorse pathologising attitudes towards homosexuality and may even have challenged such ideas, thus bucking wider heterosexist trends (Carr & Spandler, 2019). Whilst the psy disciplines continue to be a powerful institutional and discursive force in the lives of LGBTQIA+ people, our research suggests that the interaction between the psy disciplines and lesbians was more dialogical than might be assumed (see also Hegarty & Rutherford, 2019; Hubbard & Griffiths, 2019). Therefore, whilst undoubtedly the psycho-pathologisation of female-to-female relationships did stigmatise and oppress some women, it is quite possible that large numbers of lesbians and gay men lived their lives “more or less untroubled” by medical or psy-centred theories, dictates and practices (Carlston, 1997, p. 192). Our analysis also suggests that activists may have subverted psy discourse and practice, using it for their own ends, rather than merely being passive subjects of it, as critics of medicalisation, psychologisation and psychiatrisation might assume (see also Hubbard, 2020).

Finally, decisions about strategic orientations to the psy professions are still relevant to contemporary liberation struggles. The relationship between the psy professions and oppressed communities, and between different activist orientations, is often precarious and plagued with understandable mistrust and suspicion. For example, challenging the psychopathologisation and psy-centred “treatments” of trans or autistic people is a focus of current activism. Whilst there are no easy lessons to draw for psy related activism in these fields, we hope our analysis helps develop a “reparative reading” of this broader history (Sedgwick, 1997), one which avoids “presentist” analyses and supports progressive intersectional alliances across oppressed groups (Cole, 2008). In other words, it seems important to find ways to critically appreciate the activism of previous generations, however imperfect, in helping pave the way towards the liberation of all psychopathologised groups.

## Conclusion

This paper offers three main contributions. First, it analyses a lesser known history of lesbian activism in relation to the mental health system in the UK, based on an exploration of activist magazines from the 1960s–1980s. Second, it uses this history to problematise an overly reductionist or binary view of the relationship between sexuality, activism and the psy professions (i.e. that the psy disciplines are either a progressive force for social change *or* a form of oppression). Third, it suggests that grassroots magazines are an important resource for marginalised communities in supporting each other, raising awareness, sharing information and developing strategies to challenge oppression. In conclusion, whilst we highlight some limitations, or unintended consequences, of the different strategies identified, we suggest that all approaches made a distinctive contribution to challenging oppression. This suggests the benefits of strategic pluralism.

## Notes

1 The acronym LGBTQIA+ stands for lesbian, gay, bisexual, transgender, queer, intersex and asexual. It is an evolving term which attempts to be inclusive of marginalised genders and sexualities.

2 We explored various archives including: the Lesbian Archive (Glasgow Women’s Library), which includes the Lesbian Archive and Information Centre Collection (LAIC) and the Glasgow Women’s Library LGBTQ Collections, the Lesbian Information Service Archive (Lancashire Archives, Preston), The Hall-Carpenter LGBT Archives (LSE Women’s Library, London), Hall-Carpenter Oral History Archive, the London Metropolitan LGBTQ Archives (City of London Library, Guildhall), the Lesbian and Gay Foundation archives (Manchester City Library, Leeds Feminist Library North, and the Gale on-line database LGBTQ History and Culture since 1940 (Parts 1 & 2).

3 McIntosh was to go on to write the highly influential paper “The homosexual role” (McIntosh, 1968).

4 Sappho was a Greek poet from the island of Lesbos, whose name become associated with love between women.
